# Detection and follow-up of diaphragmatic dysfunction and lung parenchymal changes by ultrasound in intensive care patients receiving long-term mechanical ventilation

**DOI:** 10.5578/tt.202501974

**Published:** 2025-03-24

**Authors:** Büşra PEKİNCE, Yeşim Şerife BAYRAKTAR, Jale Bengi ÇELİK

**Affiliations:** 1 Division of Critical Care, Department of Pulmonology, Selçuk University Faculty of Medicine, Konya, Türkiye; 2 Division of Critical Care, Department of Anesthesiology and Reanimation, Selçuk University Faculty of Medicine, Konya, Türkiye

## Abstract

**ABSTRACT**

**
Detection and follow-up of diaphragmatic dysfunction and lung parenchymal changes by ultrasound in intensive care patients receiving long-term mechanical ventilation
**

**Introduction:** *Previous studies have reported that diaphragm atrophy and dysfunction might occur during mechanical ventilation (MV), but the frequency, effect on mortality, underlying causes and functional outcomes of diaphragm and lung parenchymal changes during routine MV have not yet been fully understood.
*

**Materials and Methods:** *The lung parenchyma and diaphragm of 50 patients were investigated using ultrasound (USG) on day 1, 5, and 10 of MV therapy.
*

**Results:** *Mean age of the patients was 64.90 ± 15.96 years. Mean MV duration was 90.18 ± 21.09 days. Mean thickening fraction (TFdi) on day 1, 5, and 10 was 40.77 ± 15.42, 39.85 ± 16.85, and 43.57 ± 19.10, respectively. Mean diaphragm amplitude on day 1, 5, and 10 was 1.70 ± 0.74, 1.76 ± 0.74, and 1.70 ± 0.71, respectively. Mean diaphragmatic thickness at the end of expiration (Tde) on day 1, 5, and 10 was 0.18 ± 0.08, 0.17 ± 0.06, and 0.16 ± 0.05, respectively. There was no significant change between measurement days by TFdi, diaphragmatic amplitude (DA), and Tde values. On admission, TFdi was less than 20% in 8% of the patients, DA was less than 1 cm in 12%, and Tfde was less than 0.2 cm in 52%. There was no significant difference by the TFdi, DA and lung ultrasonography (LUS) scores of the non-surviving and surviving patients. An analysis of imaging results and LUS scores indicated that LUS values were measured higher in patients with infiltration on chest radiography. In addition, LUS scores significantly decreased from day 1 to day 5 and day 10, and from day 5 to day 10.
*

**Conclusion:** *Diaphragm dysfunction may occur as a result of MV therapy or associated with an inflammatory process, including sepsis. Assessment of diaphragmatic function by USG on admission to the intensive care unit may help to better recognize and manage diaphragmatic dysfunction. LUS provides information about the lung parenchyma as important as chest X-ray and facilitates bedside patient evaluation.
*

**Key words:** *Diaphragm atrophy; mechanical ventilation; lung ultrasound; sepsis
*

**ÖZ**

**
Uzun süreli mekanik ventilasyon uygulanan yoğun bakım hastalarında diyafragma disfonksiyonunun ve akciğer parankiminde değişikliklerin ultrason ile tespiti ve takibi
**

**Giriş:** *Diyafram atrofisinin ve disfonksiyonun mekanik ventilasyon (MV) sırasında ortaya çıkabileceği yapılan çalışmalarda gösterilmiştir, ancak rutin MV sırasında diyafram ve akciğer parankimi değişikliklerinin sıklığı, mortaliteye etkisi, altta yatan nedenleri ve fonksiyonel sonuçları hakkında henüz bilgi bulunmamaktadır.
*

**Materyal ve Metod:** *Çalışmaya katılan 50 hastanın akciğer parankimi ve diyaframı MV tedavisinin birinci, beşinci ve onuncu günlerinde ultrason (USG) kullanılarak incelendi.
*

**Bulgular:** *Hastaların yaş ortalamaları 64.90 ± 15.96 yıl olarak kaydedildi. Ortalama MV süresi 90.18 ± 21.09 gün idi. Ortalama kalınlaşma fraksiyonu (TFdi) birinci günde 40.77 ± 15.42, beşinci günde 39.85 ± 16.85 ve 10. günde 43.57 ± 19.10 idi. Ortalama diyafram genliği (DG) birinci günde 1.70 ± 0.74, beşinci günde 1.76 ± 0.74 ve 10. günde 1.70 ± 0.71 olarak bulundu. Ortalama diyafragma kalınlığı ekspirasyon sonunda (Tde) birinci günde 0.18 ± 0.08, beşinci günde 0.17 ± 0.06, 10. günde 0.16 ± 0.05 idi. Ölçüm günleri arasında TFdi, DG ve Tde değerlerinde anlamlı bir değişiklik olmadı. Yatış anında hastaların %8’inin TFdi’si %20’den küçük, %12’sinin DG’si 1 cm’den küçük ve %52’sinin Tfde’si 0.2 cm’den küçük ölçüldü. Eksitus olan hastalar ile yaşayan hastaların Tfdi, DG ve akciğer ultrasonografisi (LUS) skorları arasında anlamlı bir farklılık bulunamadı. Görüntüleme bulgularının ve LUS skorlarının analizi, LUS değerlerinin akciğer grafisinde infiltrasyonu olan hastalarda daha yüksek ölçüldüğü görüldü. Ek olarak, LUS skorları birinci günden beşinci ve 10. güne ve ayrıca beşinci günden 10. güne kadar anlamlı şekilde azaldı.
*

**Sonuç:** *Diyafram disfonksiyonu MV tedavisi sonucu gelişebileceği gibi sepsis gibi enflamatuvar sürece bağlı da meydana gelebilir. Ultrason ile diyafram fonksiyonu hastaların yoğun bakım ünitesine kabulünde bakılması diyafram disfonksiyonunun daha iyi tanınmasına ve yönetilmesine yardımcı olabilir. Akciğer ultrasonografisi ise akciğer parankimi hakkında akciğer filmi kadar önemli bilgiler vermekte, yatak başında hasta değerlendirilmesinde kolaylık sağlamaktadır.
*

**Anahtar kelimeler:** *Akciğer ultrasonu; diyafram atrofisi; mekanik ventilasyon; sepsisCOVID-19; ölüm; zatürre; CT taraması
*

## INTRODUCTION

All patients undergoing mechanical ventilation (MV) in intensive care units (ICU) are considered at risk for ventilator-associated lung injury, diaphragm atrophy, and loss of function. Therefore, it is very important to monitor patients on routine MV for diaphragm atro- phy and dysfunction (1).

Intensive care patients are susceptible to develop diaphragm dysfunction associated with various factors with myotoxic effects (2,3). Sepsis, as considered one of the most important reasons for ICU admissions, is associated with diaphragm dysfunction upon altering diaphragm blood flow and contractile capacity (4,5). Histopathologic studies performed during the early period of ICU stay have reported that more than 50% of patients with sepsis had signs of neuromuscular damage (6). Furthermore, diaphragmatic dysfunction is prevalent even at ICU admission of patients who require MV procedure due to a diagnosis of respiratory failure (2,7,8).

Decreased diaphragm muscle thickness plays an important role in MV-induced diaphragm dysfunction

(9). It has been suggested that inflammation and

damage occur in the diaphragm as a result of excessive or insufficient MV support to the patient and diaphragm dysfunction might develop accordingly (10,11).

Bedside esophageal and gastric pressure measurement or electromyographic assessment of diaphragm function is challenging during MV (12). These measurements require considerable technical and physiological expertise and a level of patient cooperation that is often not available in case of intensive care patients. Furthermore, these measurements are not designed to detect changes in the diaphragmatic structure, including atrophy and paralysis that may be triggered by MV (13-16). Ultrasound (USG) is used for real-time examination of the diaphragm movement and is helpful in assessing the efficacy of MV and patient compliance.

Chest radiography is the first choice and easily accessible method for the evaluation of the lung parenchyma. Nevertheless, due to its low sensitivity and specificity, it may cause diagnostic delays (17). Therefore, lung ultrasonography (LUS) is a non- invasive, rapid, and reliable method which is an easy- to-apply technique to evaluate diaphragm mobility

and performance of respiratory muscles during MV, to detect edema, atelectasis and infiltration in the lung parenchyma early, and to follow up (18).

This study aimed to investigate diaphragm atrophy using USG in the patient population on invasive MV therapy, to understand the effects of MV therapy on lung parenchyma, to determine the effect of dia- phragm dysfunction on mortality, and to investigate the relationship between the diaphragm and the clinical status of patients at admission.

### MATERIALS and METHODS

This prospective, observational study was conducted pursuant to the World Medical Association (WMA) Declaration of Helsinki Ethical Principles for Medical Research Involving Human Participants upon approv- al by the local ethics committee and collection of informed written consent of the patients’ closest rela- tives (Approval number: E.70632468-050.01.04- 395723, date: 01.11.2022). The study included 50 patients aged between 18-90 years, who underwent invasive MV therapy with a standardized pressure support targeting a tidal volume of 6-8 mL/kg at the ICU of Selçuk University Faculty of Medicine Hospital. As per exclusion criteria, patients with neu- romuscular disease, spinal injury affecting neural conduction, known diaphragmatic dysfunction, thoracostomy, pneumothorax, pneumomediastinum, obesity (body mass index of ≥30 m^2^/kg), Stage 3-4 chronic obstructive pulmonary disease pursuant to the global initiative for chronic obstructive lung dis-

ease 2022 criteria, and pregnant patients were excluded. Furthermore, patients who did not require intubation or who died within ten days were exclud- ed from the study.

Demographic data, Sequential Organ Failure Assessment (SOFA) score, Acute Physiology and Chronic Health Evaluation (APACHE II) scores, and the occurrence of sepsis and pneumonia were prospectively recorded at ICU admission. Thirty-day mortality in the ICU, hospital mortality, and time on MV were recorded.

### Measurements

USG measurements were performed under ventilation with a tidal target of 6-8 mL/kg using synchronized intermittent forced ventilation mode (SIMV). Patients were sedated using midazolam and fentanyl to ensure a Riker Sedation-Agitation Scale score of 3-4. Lung parenchyma and diaphragm were examined by USG on the first, fifth and tenth day of MV treatment. Diaphragmatic evaluation by USG was performed using a linear probe (L12-4 MHz, Mindray DC-60) (Figure 1). Diaphragmatic thickness was measured at end-expiration (Tde) and at end-inspiration (Tdi), and the thickening fraction (TFdi) was calculated offline as (Tdi-Tde)/Tde×100. Diaphragmatic amplitude (DA) was measured in motion (M) mode using a convex probe (C4-2 MHz, Mindray DC-60) (19-21) (Figure 2). USG measurements were performed in at least three separate respiratory cycles, and the mean value of the three measurements was recorded.

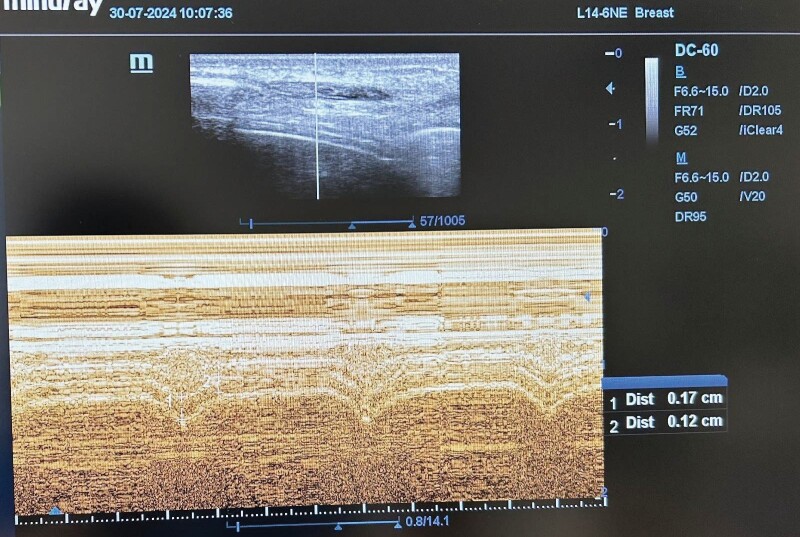

**Figure 1.** Diaphragmatic thickness was measured Tde and TFdi.

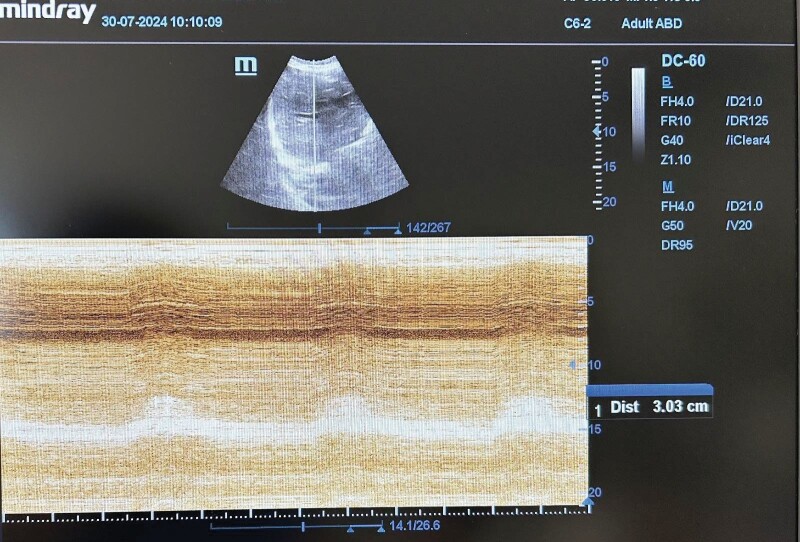

**Figure 2.** Diaphragmatic amplitude measured in motion (M) mode.

The Tde <0.2 cm, DA <1 cm, and TFdi <20% thresholds as suggested by previous studies were used in the measurements of diaphragm dysfunction (20-22).

A convex probe was used for lung parenchymal evaluation. Each lung was first divided into anterior and lateral regions, and then each region was divided into superior and inferior regions, taking the anterior axillary line as a landmark. Measurements were evaluated by an intensive care physician experienced in LUS.

The following scoring criteria were used for evaluation (23).

Score 0: Lines A, sliding with respiratory movement, two or less lines compared to B lines,

Score 1: Three or more B lines,

Score 2: Multiple combination of B lines,

Score 3: Occurrence of pulmonary consolidation or tissue pattern. The sum of the scores from the anterior and lateral regions was recorded as the anterolateral score (ranging from 0 to 16).

### Statistical Analysis

Statistical software language R version 4.1.2 (www.r- project.org) was used for all statistical analyses included in the study. Before the analyses, normal distribution hypothesis for the study data was tested by Shapiro-Wilk normality test and the homogeneity

of group variances was tested by the Levene’s test. Numerical variables were presented as mean ± standard deviation or median with minimum and maximum values, where categorical variables were presented as frequency (n) and percentage (%). Repeated measures analysis of variance was used to analyze whether there was a significant change in TFdi, DA, and LUS values between the days of measurement, and Bonferroni corrected dependent sample t-test results were used for pairwise comparisons between the times for the variables with significant change. Furthermore, student’s t-test, Welch’s t-test and Mann-Whitney U test were used to investigate whether there was a significant difference in LUS measurements vis-a-vis the imaging results. Furthermore, the TFdi, DA, and LUS scores of the non-surviving and surviving patients were compared using student’s t-test and Welch’s t-test. Student’s t-test and Welch’s t-test were used to compare the scores by diagnoses on admission. Significance level was taken as 5%.

### Sample Size Calculation

A preliminary power analysis was performed to detect a minimal difference in TFdi, DA and LUS values within measurement times (within groups design) at a significance level of 5%, statistical power of 95%, and 0.25 effect size using a two-tailed repeated measurement analysis of variance test. The minimum required sample size was 43, but

**Table:** 

**Table 1.** Patient characteristics	
**Patients (n= 50)**	
Age (years)	64.90 ± 15.96 (25-89)
18-64/64-90	19 (38)/31 (62)
Sex (M/F)	24 (48)/26 (52)
Mortality	36 (72)
Length of stay on mechanical ventilation	90.18 ± 21.09 (11-490)
Extubation status at the end of ten days	5 (10)
Tracheostomy status at the end of ten days	45 (90)
Comorbidity	
Hypertension	25 (50)
Diabetes	16 (32)
Coronary artery disease	13 (26)
Cerebrovascular disease, epilepsy, Alzheimer	13 (26)
Chronic obstructive pulmonary disease	8 (16)
Malignity	5 (10)
Other disease	3 (6)
Hospitalization diagnoses	
Pneumonia	13 (26)
Sepsis	10 (20)
Cerebrovascular disease	13 (26)
Trauma	6 (12)
Myocardial infarction	8 (16)
M/F: Male/Female.

considering a drop-out rate of 10% in relevant research, 50 patients were included in this study.

## RESULTS

Fifty patients (24 males and 26 females) on MV therapy were included in the study. Nineteen patients were aged between 18-64 and 31 patients were between 64-90 years. Patients were admitted to the ICU with a diagnosis of pneumonia (26%), cerebro- vascular disease (26%), myocardial infarction (MI) (16%), trauma (12%), and sepsis (20%). Five patients were extubated after ten days of follow-up. Forty patients underwent tracheostomy. The patients’ mean duration of MV was 90.18 ± 21.09 days. Hospital mortality was 72% (Table 1).

While 30-day mortality was significant with the increase in APACHE score, there was no statistically significant correlation between SOFA and Tde, TFdi and DA measurements on day 1, 5, and 10 (Table 2). There was no significant difference between the TFdi, DA and LUS scores of the non-surviving and surviving patients (Table 3).

Mean TFdi on day 1, 5, and 10 was 40.77 ± 15.42,

39.85 ± 16.85, and 43.57 ± 19.10, respectively.

Mean DA on day 1, 5, and 10 was 1.70 ± 0.74, 1.76

± 0.74, and 1.70 ± 0.71, respectively. Mean Tde on day 1, 5, and 10 was 0.18 ± 0.08, 0.17 ± 0.06, and

0.16 ± 0.05, respectively. There was no significant change between the days of measurement by TFdi, DA, and Tde values (Table 4).

On admission, TFdi was less than 20% in 8% of the patients, DA was less than 1 cm in 12%, and Tde was less than 0.2 cm in 52% of the patients (Table 5).

Upon an analysis of the measurements were analyzed according to the diagnosis on admission, DA was lower on day 10 compared to the first day (1.26 ± 0.29 vs. 1.79 ± 0.73, p= .002) only in patients with MI.

As a result of an analysis of imaging findings and LUS scores, LUS values were higher in patients with infiltration on chest radiography. There was also a significant decrease in LUS scores from day 1 to day 5 and day 10, and from day 5 to day 10. Mean LUS

score on day 1, 5, and 10 was 14.14 ± 8.36, 11.58 ±

6.93, and 9.64 ± 5.55, respectively (Table 6).

## DISCUSSION

MV is considered one of the fundamental treatment modalities in the ICU where potential complications should be closely monitored. Results of the present study suggested the importance of USG as a non- invasive tool to evaluate prevalent complications and to monitor lung parenchyma and diaphragm atrophy in patients on MV therapy. Accordingly, patients

**Table:** 

**Table 6.** Lung ultrasound score results by imaging findings								
**LUS measurements**								
**n Mean ± SD Median (range) p**								
Chest radiography	Abnormal	43	16.21	±	7.05	16	(3-30)	<0.0011
	Normal	7	1.43	±	1.90	0	(0-4)	
Infiltration	None	25	11.56	±	8.99	10	(0-30)	0.0282
	Yes	25	16.72	±	6.94	16	(3-30)	
Atelectasis	None	47	13.77	±	8.40	14	(0-30)	0.1761
	Yes	3	20.00	±	6.00	20	(14-26)	
Pleural effusion	None	43	13.63	±	8.62	14	(0-30)	0.2882
	Yes	7	17.29	±	6.07	20	(10-26)	
Pulmonary edema	None	42	14.12	±	8.29	14	(0-30)	0.9682
	Yes	8	14.25	±	9.33	13	(5-30)	
1Mann-Whitney U test, 2Student’s t-test, 3Welch’s t-test. LUS: Lung ultrasonography, SD: Standard deviation.

might have diaphragmatic dysfunction at ICU admis- sion and 30-day mortality was higher in patients with higher APACHE II scores. There was no decrease in TFdi, DA and Tde at ten-day follow-up of the patients, but there was a significant decrease in LUS score. Furthermore, TFdi and DA first decreased and then increased in some patients. A study of 107 patients by Goligher et al. reported changes in diaphragm thickness occurred in the early period in patients who underwent a seven-day MV procedure and also indicated an increase in DA in some patients (18). Goligher suggested that both decreased and increased diaphragm thicknesses were associated with signifi- cant diaphragm dysfunction.

It is challenging to plan MV therapy for the elderly population, which has a high mortality rate associated with advanced age and the occurrence of prevalent geriatric symptoms, including frailty, cognitive decline, and multiple comorbidities. In the present study, mean age was 64.90 ± 15.96 years and there were 31 patients over 65 years of age. Tde was <0.2 cm at ICU admission in 17 patients with advanced age. A study by Er et al. suggested that USG could be used to monitor diaphragm dysfunction at the termination of MV treatment in elderly patients (24).

There are multiple risk factors for diaphragmatic dysfunction in ICU patients. The first is associated with the existing diseases of the patients at the time of ICU admission (25). Similar to all striated muscles, dysfunction may occur in the diaphragm as a result of shock-related organ failure, which is observed in many patients at admission to the ICU and in which sepsis plays an important role (26). Furthermore, mediators released during sepsis may induce a decrease in the contractile capacity of the diaphragm

(5). Fifty percent of the patients were followed up with a diagnosis of pneumonia and sepsis in the present study. Therefore, the low TFdi, DA, and Tde measured at admission might be due to diaphragmatic dysfunction secondary to sepsis. We think that the diagnosis of sepsis was a major independent risk factor for diaphragmatic dysfunction at ICU admission in our patients, which was consistent with the animal data of Hussain et al., who associated sepsis with early-onset diaphragmatic dysfunction (27).

MI disease group is typically followed up at the ICU, and MV therapy is introduced as necessary. In a prospective study, Spiesshoefer et al. suggested upon USG examinations that increased circulating IL-6 and TNF-*α* levels in patients followed up for MI may

be associated with respiratory muscle dysfunction

(28). A study by Aubier et al. suggested that diaphragm dysfunction occurred as a result of cardiogenic shock

(29). In seven out of our eight patients admitted to the ICU with a diagnosis of MI, Tde values measured on admission were less than 2 mm. We also found that the DA was lower on day 10 compared to the day 1 of the ten-day follow-up of our patients. Accordingly, we thought that in cases where hypotension caused by cardiogenic shock leads to organ failure, the diaphragm might act like an organ and the loss of function might occur secondary to hypotension.

Cerebrovascular events (CVE) are a group of diseases marked with high morbidity and mortality and frequently followed up at the ICU (30). As a result of stroke, respiratory failure may occur due to the involvement of respiratory control centers or weakness of respiratory muscles, and consequently, the need for MV may arise (31). Ripoll et al. reported in a prospective study of 60 patients with supratentorial ischemic stroke that the possibility of diaphragm dysfunction was high within 48 hours upon measuring Tdfi with USG (32). Consistently, in the present study, we measured low Tde in 5 out of 13 patients with CVE on admission and in correlation with this, we opened tracheostomy in 12 patients as a result of prolonged intubation. We think that diaphragmatic dysfunction, which is one of the respiratory system complications that may develop in patients with CVE, should be closely monitored using USG.

Another risk factor for diaphragmatic dysfunction in patients admitted to the ICU is prolonged MV (33). Vassilakoulos and Petrof hypothesized in an animal study that a loss of force occurs in the diaphragm after initiation of ventilation (33). Upon comparison of the diaphragm biopsies of 14 organ donor patients with brain death and eight patients who underwent thoracic surgery, Levine et al. reported that the combination of 18-69 hours of complete diaphragmatic inactivity and MV led to marked atrophy in diaphragm myofibrils (13).

Many animal experiments suggested that atrophy developed due to nonuse of the diaphragm (34-36). Nevertheless, the importance of nonuse-related atrophy in the clinical setting is unclear because assisted ventilation modes are widely used (37,38). Most patients show minimal diaphragmatic effort in assisted mechanical ventilator modes. It will require little or no diaphragm effort with low levels of auxiliary muscle activation or automatic triggering,

given the sensitivity of ventilator triggering (39). In the present study, there was no significant change in the measured TFdi, Tde, and DA values of our patients after ten days of MV treatment. We think that this might be related to the fact that our patients were on SIMV mode, and the lungs were passively ventilated, so mechanical displacement of the diaphragm was possible.

Previous studies suggested that diaphragm weakness greatly prolonged the time required to wean patients from MV and worsened clinical outcomes (40). Furthermore, Jung et al. suggested that diaphragm dysfunction was an important cause of ICU mortality by measuring TFdi with USG (8). In the present study, there was no association between diaphragmatic dysfunction at the beginning of MV or during subsequent ICU stay and mortality in our patients. We attributed this to the heterogeneity of the diagnoses at admission and chronic diseases of our patients. Furthermore, the length of stay in MV was

90.18 ± 21.09 days, and 40 patients underwent tracheostomy secondary to prolonged intubation. This result is consistent with that of Goligher et al. We think that it is compatible with their study on ICU patients in which they suggested that the risk of tracheostomy increased due to prolonged MV duration because of diaphragm atrophy (10).

LUS has become an important tool for early bedside diagnosis of prevalent complications of MV, including pleural effusion, atelectasis or alveolar consolidation due to ventilator-associated pneumonia and pneumothorax (41,42). A review by Jinwoo Lee suggested that bedside LUS, especially in an ICU setting, was more advantageous compared to chest radiography (43). The LUS values were higher in our patients consistent with their chest X-rays on admission. The significant decrease in the LUS score of our patients at follow-up was correlated with improvement in the routine chest X-rays during their treatment. Lung parenchyma was improved upon medical treatment, especially in our patients who were followed up for pneumonia and pulmonary edema. Nevertheless, despite the decrease in LUS score, there was no improvement in the diaphragm function in our patients. We think that diaphragmatic dysfunction may affect lung function even when lung USG is normal, and this may play a critical role in respiratory support and other treatment approaches.

A study by Abdullayeva et al., which investigated the effect of interstitial lung diseases on lung parenchyma

with USG, suggested that USG can be considered as promising, new, non-invasive tool, especially when combined with chest radiography (44). We recommend LUS for early diagnosis and close follow- up of changes in the lung parenchyma associated with the effect of MV during respiratory failure in our patients.

The limitations of the present study include the relatively smaller number of patients, its single-center design, the fact that the patients were in the advanced age group, and that the diagnosis on admission to ICU and chronic disease history varied.

## CONCLUSION

We suggest that diaphragmatic dysfunction should be considered as an organ dysfunction that may be indicative of a serious underlying disease and that acute diaphragmatic insufficiency should be detected at ICU admission. We emphasize the importance of monitoring patients on MV based on LUS scores and identifying risk factors for diaphragmatic dysfunction based on comorbidity and diagnoses at admission.

We remind that USG is an inexpensive, noninvasive, radiation-free and bedside method for assessing diaphragm thickness and lung parenchyma during MV procedure.

Finally, more comprehensive multicenter studies with a customized patient population are required in the future to evaluate diaphragm function changes in ICU patients on MV on the basis of diagnoses at admission and LUS scores and to understand the clinical consequences of these changes.

**Ethical Committee Approval:** This study was approved by Selçuk University Faculty of Medicine Ethics committee (Approval number: E.70632468- 050.01.04-395723, Date: 01.11.2022).

### CONFLICT of INTEREST

The authors declare that they have no conflict of interest.

